# Positive Emotion Regulation in Addiction: A New Frontier for Recovery Science

**DOI:** 10.1007/s40429-026-00741-3

**Published:** 2026-04-10

**Authors:** Eric L. Garland

**Affiliations:** 1 Department of Psychiatry, University of California San Diego, CA La Jolla, USA; 2 Sanford Institute for Empathy and Compassion, University of California San Diego, CA La Jolla, USA

**Keywords:** Emotion regulation, Positive affect, Reward, Savoring, Substance use disorder

## Abstract

**Purpose of Review:**

Addiction has traditionally been framed as a disorder of negative affect and impaired downregulation of distress, yet disturbances in positive emotional experience and regulation are equally fundamental to the development and persistence of substance use disorders (SUDs). This review synthesizes clinical and psychophysiological evidence documenting the impact of addictive substances on positive emotion dysregulation, and highlights the role of positive emotion regulation on craving and drug relapse.

**Recent Findings:**

Individuals who engage in addictive use of opioids, nicotine, cannabis, and stimulants exhibit blunted positive affect, diminished responsiveness to natural rewards, and deficits in the volitional upregulation of positive emotion. These impairments reflect neuroplastic alterations in corticostriatal and corticolimbic circuits that weaken endogenous reward generation, and erode the capacity to derive pleasure from, and savor, everyday experiences.

**Summary:**

Addiction is underpinned by positive emotion dysregulation. Restoring positive affective functioning through positive emotion regulation interventions may revolutionize addiction recovery.

## Introduction

Traditional models of addiction emphasize negative reinforcement: individuals use substances to blunt distress, escape dysphoria, or regulate aversive internal states [1, 2]. While this framework captures an important dimension of addictive behavior, it overlooks a parallel and equally consequential process that strikes at the heart of the pathogenic mechanisms driving addiction: dysregulation of positive emotional experience. Emerging evidence suggests that addiction involves not only heightened negative affect and difficulties regulating it, but also fundamental deficits in responsiveness to and enjoyment of natural rewards and positive emotions. These deficits contribute to anhedonia, craving, and compulsive drug use among people with substance use disorders (SUDs), and may undermine addiction recovery by limiting access to natural sources of reward and well-being.

The aim of the current review is to synthesize evidence on positive emotion dysregulation in addiction, drawing on clinical studies, neuropharmacology, psychophysiological research, and neuroimaging findings. The review then evaluates interventions designed to strengthen positive emotion regulation among people with SUDs and summarizes the evidence for their efficacy and underlying mechanisms.

## Neurobiological Mechanisms Underlying Positive Emotion Dysregulation

Across various psychoactive substance use, addiction-related deficits in positive emotion arise from neuroplastic changes within cortico-striatal and cortico-limbic circuits that govern reward processing, motivation, and affective experience. Chronic exposure to addictive substances disrupts dopaminergic signaling in the ventral striatum and alters communication between the ventral tegmental area, nucleus accumbens, amygdala, and prefrontal cortex, regions essential for encoding the salience, anticipation, and enjoyment of natural rewards [3]. These drug-induced neuroadaptations include downregulation of dopamine receptor availability, reduced striatal responsivity to non-drug rewards, and impaired prefrontal top-down modulation of reward signals [4], changes that collectively contribute to anhedonia and blunted positive affect. Over time, repeated substance use drives an allostatic shift in reward circuitry in which drug cues acquire exaggerated motivational value while natural rewards lose their capacity to evoke pleasure, reinforcing compulsive drug seeking and impairing the ability to upregulate positive emotional states [5]. Synaptic plasticity within these circuits, including alterations in glutamatergic transmission and maladaptive strengthening of drug-associated learning, further entrenches this imbalance, biasing behavior toward drug-related reinforcement at the expense of healthy reward processing [6]. Together, these neurobiological changes form a core transdiagnostic mechanism through which addiction undermines positive emotion regulation and perpetuates substance use. In the sections that follow, specific neurobiological mechanisms of positive emotion dysregulation are reviewed for opioids, nicotine, and other drugs of abuse (cannabis, cocaine, methamphetamine).

## Blunted Positive Affect and Reward Responsiveness in Addiction

### Opioid-Related Neuroadaptations to Reward Circuitry

Exogenous opioids exert their effects by binding to and dysregulating endogenous µ-opioid receptor (MOR) systems that modulate hedonic tone and reward valuation [7–10]; chronic activation of MORs leads to downregulation, altered receptor signaling, and impaired endogenous opioid tone [11–13]. At the neuropharmacological level, opioids inhibit GABAergic interneurons in the ventral tegmental area, disinhibiting dopamine neurons and thereby increasing dopamine release in the nucleus accumbens, a mechanism central to opioid reinforcement and reward [14, 15]. Chronic opioid exposure produces a constellation of neuroplastic alterations within cortico-striatal and cortico-limbic circuits that are central to reward processing. Long-term opioid use is associated with reduced dopamine D2 receptor availability in the striatum, a hallmark of impaired reward signaling [16]. Structural neuroimaging studies further demonstrate decreased gray matter volume in regions supporting reward and affective regulation, including the amygdala and orbitofrontal cortex, following sustained opioid administration [17]. Opioid dependence is also linked to weakened functional connectivity between limbic and striatal structures, reflecting disruptions in the integration of emotional and motivational information [18]. Additional work has identified less robust and more restricted value representations in the ventromedial PFC and throughout limbic and salience networks among people with opioid use disorder (OUD) [19]. Together, these neurobiological adaptations undermine the brain’s capacity to encode, anticipate, and respond to natural rewards, contributing to anhedonia, diminished positive affect, and escalating drug use.

### Evidence of Blunted Positive Affect and Reward Responsiveness in Opioid Misuse and Opioid Use Disorder

An emerging body of research demonstrates that individuals who misuse prescription opioids or meet diagnostic criteria for OUD exhibit anhedonia [20, 21] and marked reductions in positive emotional responding [17, 22–24]. Relative to healthy controls and opioid-treated patients who use medications as prescribed, opioid misusing individuals and those with OUD show attenuated positive affect when viewing pleasant stimuli [25], diminished parasympathetic flexibility during positive emotional processing [23], reduced attentional engagement with rewarding cues [26], and blunted electrocortical [27–29] and prefrontal blood flow responses to natural rewards [30–32]. These abnormalities persist even after accounting for chronic pain, suggesting that opioid exposure itself contributes to dampening of reward system function. Importantly, reduced positive affective reactivity predicts greater opioid craving [23] and promotes relapse [27], indicating that anhedonia may motivate compensatory drug use. This finding in congruent with allostatic models [33–36] suggesting that opioid misuse drives insensitivity to natural rewards, which in turn impels craving as opioids as a means of obtaining hedonic equilibrium.

### Nicotine-Related Neuroadaptations to Reward Circuitry

Nicotine addiction produces a pattern of neurobiological alterations that closely parallels the reward-system disruptions observed in other substance use disorders. Nicotine binds to α4β2 nicotinic acetylcholine receptors (nAChRs) on dopaminergic neurons in the ventral tegmental area, producing phasic dopamine release in the nucleus accumbens, a core mechanism underlying its reinforcing effects [37, 38]. Nicotine also modulates glutamatergic and GABAergic inputs to midbrain dopamine neurons, enhancing excitatory drive and reducing inhibitory tone, which further amplifies reward-related dopamine signaling [39]. Chronic nicotine exposure leads to upregulation and desensitization of nAChRs, producing neuroadaptations that sustain dependence and alter reward sensitivity [40]. Consequently, chronic nicotine exposure is associated with structural alterations in frontostriatal and limbic regions, including reduced gray-matter volume in regions of prefrontal cortex, anterior cingulate, and insular cortex, reflecting long-term changes in circuits that regulate reward [41–43]. Nicotine dependence is also linked to disrupted functional connectivity within mesocorticolimbic networks, particularly weakened coupling between the prefrontal cortex, striatum, and insula, which predicts craving intensity and relapse vulnerability [44–46]. Longitudinal imaging studies further show that chronic smokers who experience more relapse during treatment exhibit aberrant connectivity and altered network topology in salience and default-mode networks, indicating widespread neuroadaptations that bias attention, valuation, and reward processing toward nicotine [47].

### Evidence of Blunted Positive Affect and Reward Responsiveness in Nicotine Addiction

A pattern of blunted positive emotional responding is evident in nicotine addiction. Neuroimaging studies consistently show that smokers exhibit reduced electrocortical [48–51] and striatal activation to non–drug-related rewards [43], diminished prefrontal engagement during reward anticipation [52, 53], and weaker neural responses to positive emotional cues [49]; these impairments in reward processing are clinically meaningful: blunted responsiveness to natural rewards reliably predicts smoking lapses and relapse. Chronic smoking is associated with blunted striatal responses to non–drug rewards [54], reflecting reduced sensitivity to natural reinforcers and diminished dopaminergic signaling within the ventral striatum [55]. Neuroimaging studies further demonstrate dysregulated prefrontal control during affective and reward-related processing, including attenuated activation in dorsolateral and ventromedial prefrontal regions during reward anticipation and valuation [52]. Moreover, nicotine withdrawal further intensifies positive affective blunting [56], producing an anhedonic state in which everyday rewards feel muted or unrewarding. This diminished capacity to experience pleasure from naturally rewarding stimuli may increase reliance on nicotine as a means of restoring hedonic tone, thereby perpetuating the cycle of addiction. Collectively, these findings indicate that nicotine addiction involves a weakening of the neural systems that support positive emotional experience, reducing responsiveness to natural rewards.

### Evidence of Blunted Positive Affect and Reward Responsiveness in Other Drugs of Abuse

Similar disturbances in positive affect and reward responsiveness have been documented for other commonly misused substances, including cannabis, cocaine, and methamphetamine. Chronic cannabis use is associated with anhedonia [57] and reduced striatal dopamine synthesis capacity and blunted dopamine release [58, 59], indicating hypodopaminergic function within mesolimbic reward pathways [60]. A recent systematic review of brain reward function in people who use cannabis indicates that, across studies, heavy or chronic cannabis use is frequently associated with reduced activation in striatal and prefrontal regions during anticipation or receipt of non–drug rewards and with blunted subjective responses to naturally rewarding stimuli [61]. These alterations in reward circuitry appear more pronounced with earlier onset, greater frequency, and higher cumulative exposure, suggesting dose-dependent dampening of natural reward responsiveness. Cocaine and methamphetamine use show a comparable pattern of reward system disruption. Both substances acutely amplify dopaminergic signaling in mesocorticolimbic pathways [62], but with repeated use they induce neuroadaptations that blunt dopamine release [63], biasing the system toward drug cues and away from natural rewards [64]. Chronic stimulant users often display attenuated striatal and orbitofrontal responses to monetary and social rewards [62, 65], alongside diminished positive affect and anhedonia [66, 67], and these deficits predict poorer treatment outcomes and higher relapse risk. Converging evidence thus suggests that like opioids and nicotine, cannabis, cocaine, and methamphetamine progressively hijack and blunt the brain’s capacity to generate and sustain positive emotional responses to everyday, healthful sources of reward.

## Deficits in Volitional Upregulation of Positive Emotion

Positive emotion dysregulation in addiction extends beyond blunting of reactive affective responses. Individuals with SUD also frequently struggle to proactively generate, enhance, or sustain positive emotional states, reflecting impairments in the cognitive and neural mechanisms that support volitional positive emotion regulation. Deficits in proactive upregulation of positive emotion reflect broader impairments in prefrontal regulatory systems responsible for sustaining attention to positive stimuli, modulating reward responses, and integrating emotional information [68]. These prefrontal deficits are theorized to impair the ability to maintain attention on positive cues, inhibit competing negative or drug-related stimuli, and effectively amplify natural reward responses by modulating striatal circuitry [69]. When these regulatory systems are compromised, individuals become less able to derive pleasure from natural rewards and more susceptible to the motivational pull of drug cues. This imbalance reinforces the cycle of addiction by narrowing the emotional repertoire and reducing access to adaptive, positive affective states.

A growing body of evidence indicates that individuals who misuse opioids exhibit profound impairments in their ability to volitionally upregulate positive emotion via *savoring*, a positive emotion regulatory strategy involving focusing attention on the pleasant sensory, affective, and cognitive features of natural rewards as a means of increasing positive emotion and pleasure derived from the rewarding experience [69, 70]. Chronic pain patients who misuse prescription opioids show reduced sympathetic arousal [71], blunted heart rate variability responses, and diminished capacity to increase positive affect via savoring [23]. Higher opioid doses are associated with lower ability to amplify positive emotional responses during savoring [23], suggesting dose-dependent disruption of prefrontal and reward-related circuitry involved in positive emotion regulation.

**Fig. 1:**
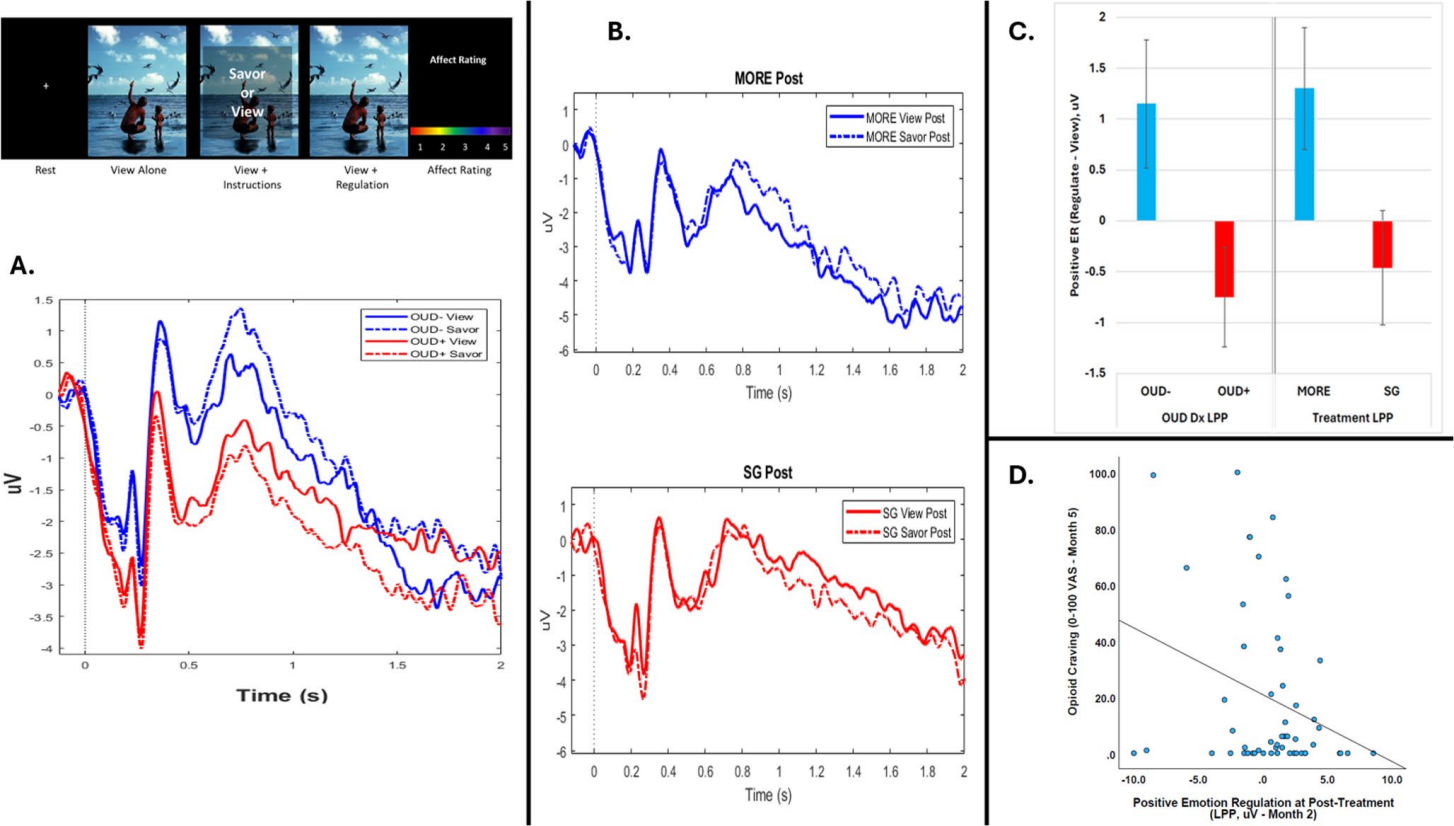
Positive emotion dysregulation in opioid use disorder (OUD) and remediation by mindfulness-oriented recovery enhancement (MORE). 1**A**) Patients with OUD (OUD+) demonstrated deficits in proactive upregulation of response to positive affective stimuli relative to those without OUD (OUD-), as indicated by the late positive potential (LPP) of the EEG. The group (OUD + vs. OUD-) × strategy (savor vs. view) interaction was significant: B = 1.91; 95% CI, 0.85–2.96; *P* < .001. 1**B**) Eight weeks of MORE remediated this deficit, whereas supportive group (SG) therapy did not. The treatment (MORE vs. SG) X strategy (savor vs. view) interaction was significant: B = 1.53; 95% CI, 0.33–2.73; *P* = .01. 1**C**) MORE normalized the positive emotion regulation deficit in OUD. The positive emotion regulation LPP response of patients treated with MORE was similar to that of people without an OUD diagnosis. 1**D**) Higher positive emotion regulation LPP activity at post-treatment predicted lower opioid craving at post-treatment, B = − 2.38; 95% CI, − 4.55 to − 0.21; *P* = .03. Data from Garland et al. [24]

Some of the strongest evidence for impaired positive emotion regulation in OUD comes from a recent mechanistic study published in *JAMA Psychiatry*. In a secondary analysis of a randomized clinical trial, Garland et al. [24] demonstrated in a sample of 160 people who use opioids that those with OUD exhibit profound deficits in their ability to volitionally upregulate positive emotion. During a validated positive emotion regulation task, patients with OUD showed markedly reduced P300 and late positive potential (LPP) amplitude when attempting to savor positive stimuli, reflecting diminished salience detection and reduced motivated attention to natural rewards. Unlike patients treated with opioid analgesics who did not meet criteria for OUD, those with OUD failed to increase neural responses during savoring (Fig. 1a). This OUD-related deficit in savoring capacity was significantly associated with higher levels of opioid craving. These findings provide direct evidence that OUD disrupts the capacity to intentionally amplify positive emotional responses.

Recent neuroimaging work helps clarify the broader corticostriatal mechanisms that may underlie these LPP abnormalities. In a large fMRI study of individuals with OUD, Huang et al. [72] found heightened nucleus accumbens, orbitofrontal cortex, and ventromedial prefrontal cortex reactivity to drug cues, coupled with blunted engagement of these same regions during savoring of images representing non-drug natural rewards (e.g., food). Individuals with heroin addiction also showed reduced rostral anterior cingulate cortex activation during savoring, and greater reliance on dorsolateral prefrontal cortex during drug cue reappraisal, patterns that were strongly associated with craving and treatment duration. These findings indicate that OUD involves a fundamental imbalance in cortico-striatal systems: hyper-reactivity to drug cues and hypo-reactivity to natural rewards, a neural functional pattern that is consistent with the LPP blunting to natural rewards observed by Garland et al. [24].

Proactive positive emotion regulation has been studied less for other substances. Smokers report difficulty enhancing positive affect and often rely on nicotine to artificially induce pleasurable states, reflecting a compensatory strategy for underlying deficits in endogenous reward generation [73, 74]. However, to date there have been no studies of the impact of smoking on the capacity to proactively upregulate responses to positive emotional stimuli. Deficits in savoring are associated with cannabis use problems and mediate cannabis use on cannabis use problems [75, 76]. Dampening positive affect (down-regulating positive affect) was positively associated with general substance use among people in SUD treatment who had higher levels of pre-treatment drug use [77]. Although far more research on positive emotion dysregulation in other substances is needed, these findings suggest that impairments in savoring are not substance-specific but represent a broader transdiagnostic mechanism through which addiction erodes the capacity for healthy positive emotion regulation.

## Clinical Implications and Treatment Targets

Positive emotion dysregulation is not a secondary or downstream consequence of addiction, it is a core driver of the disorder. Across substances, chronic drug use reshapes reward circuitry in ways that blunt natural pleasure, weaken the capacity to generate or sustain positive emotional states, and narrow the emotional repertoire to drug-related sources of reward. As positive affective functioning erodes, individuals become increasingly reliant on substances to preserve a dwindling hedonic tone, creating a downward spiral in which anhedonia fuels craving, craving fuels compulsive use, and compulsive use further deepens reward system dysfunction [35, 78]. This dynamic helps explain why many individuals continue using despite diminishing pleasure from the drug itself: the drug becomes the only reliable means of escaping a pervasive deficit in positive emotion.

As such, interventions that strengthen positive affective functioning offer a potential pathway for interrupting this cycle. By restoring sensitivity to natural rewards, enhancing the ability to upregulate positive emotion, and broadening access to sources of pleasure and meaning beyond the drug, these approaches may counteract the hedonic deficits that sustain addictive behavior. According to the *restructuring reward hypothesis*, increasing responsiveness to natural healthy rewards through proactive up-regulation of positive emotions will decrease the relative salience of drug-related rewards, and thereby decrease craving and addictive behavior [69, 79, 80]. Strengthening positive emotion regulation thus represents not only a therapeutic adjunct but a mechanistic target potentially capable of shifting the trajectory of addiction toward recovery.

The efficacy of emotion regulation-focused interventions in general has been assessed meta-analytically and shown to significantly reduce substance use [81]. Comparatively less is known about addiction treatments focused specifically on positive emotion regulation. Positive psychological interventions involving savoring, positive affect induction, and cultivating gratitude toward positive daily events have demonstrated promise in the treatment of substance use. For instance, studies of such interventions have shown significant effects on smoking craving [82] and smoking frequency [83], cannabis use [84], and methamphetamine use [85]. However, across the small body of randomized controlled trials (RCTs) of positive psychological interventions, a recent meta-analysis found nonsignificant effects on substance use-related outcomes (d = 0.11) [86].

In contrast, Mindfulness‑Oriented Recovery Enhancement (MORE), an intervention that integrates intensive and targeted training in mindfulness and savoring techniques to restore natural reward processing and enhance positive emotion regulation, shows robust and replicated statistically significant effects on substance use and craving. Multiple RCTs in patients with opioid misuse and OUD demonstrate that MORE increases autonomic (e.g., cardiac and galvanic skin response) and electrocortical responses (e.g., LPP) to natural rewards [87–91], improves positive affect [92–95], and reduces craving and substance use [95–98]. Recent neurophysiological evidence provides the strongest mechanistic support to date: in a mechanistic substudy of a randomized clinical trial, Garland et al. [24] showed that MORE normalized neural deficits in positive emotion regulation among people with OUD, producing significant increases in LPP amplitude during savoring relative to a supportive therapy control (see Fig. 1b & c). MORE also enhanced attention to positive information, increased positive affect, and reduced anhedonia. Although the underlying neural generators of this effect remain to be ascertained, a recently completed neuroimaging RCT in people with heroin addiction found that MORE normalized the hyper-reactivity to drug cues and hypo-reactivity to natural rewards [99] observed among people with OUD [72].

Complementing this opioid-focused work, evidence from nicotine addiction further supports the role of savoring-based interventions in restructuring reward processes. In a pilot quasi-experimental fMRI study of cigarette smokers, Froeliger et al. [100] found that MORE produced increased ventral striatal and ventromedial prefrontal cortex activation during positive emotion regulation, concomitant with reduced cue-reactivity in these same regions. MORE also strengthened resting-state functional connectivity between the orbitofrontal cortex and rostral anterior cingulate cortex, brain structures involved in reward valuation and self-regulation. These neural changes were strongly correlated with reductions in smoking behavior and increases in positive affect, indicating that MORE restructures reward responses to diminish the motivational pull of cigarette cues.

In my appraisal of this body of research, the aforementioned studies of MORE provide strong support for the restructuring reward hypothesis, demonstrating across multiple replications, diverse measurement modalities, and independent RCTs that increases in positive affective processes and psychophysiological responsiveness to natural rewards are key mechanisms driving reductions in craving (Fig. 1d) and substance misuse [87, 88, 91–93, 99, 100]. These mechanistic findings point to a potential breakthrough in addiction treatment: interventions that restore positive emotion regulation may directly counteract the hedonic deficits that drive addictive behavior and support long‑term recovery. Whereas prior generations of addiction treatments have largely focused on reducing aversive experience—dampening stress, suppressing craving, or avoiding triggers—this emerging approach suggests that enhancing positive affective experience can directly target the reward‑system dysfunction that characterizes addiction. By rebuilding the capacity to experience natural pleasure, such interventions may help restore hedonic function and reverse the allostatic shift toward drug‑centered reinforcement. Future integrated interventions combining cognitive-affective training (e.g., MORE) with neuromodulation (e.g., neurofeedback or stimulation of corticostriatal circuitry) and/or novel pharmacotherapies may be especially potent in that regard.

## Conclusion

Positive emotion dysregulation represents a fundamental, yet historically underappreciated, dimension of addiction. Blunted positive affect, impaired reward responsiveness, and deficits in volitional upregulation of positive emotion contribute to craving and addictive behavior. Neurobiological evidence underscores the centrality of reward system dysfunction in these processes. Interventions that restore positive affective functioning offer a potential revolution in addiction treatment development. Targeting positive emotion dysregulation may be an essential therapeutic process in addiction recovery by helping individuals shift from drug-centered reward processing toward a more balanced, naturally rewarding, and meaningful life.

## Key References

- Carrico AW, Neilands TB, Dilworth SE, Evans JL, Gόmez W, Jain JP, et al. Randomized controlled trial of a positive affect intervention to reduce HIV viral load among sexual minority men who use methamphetamine. J Intern AIDS Soc. 2019;22:e25436. https://doi.org/10.1002/jia2.25436.
  - ○ Evidence from a robust trial demonstrating that a positive affect intervention can reduce methamphetamine use.
- Garland EL, Hudak J, Hanley AW, Bernat E, Froeliger B. Positive Emotion Dysregulation in Opioid Use Disorder and Normalization by MindfulnessOriented Recovery Enhancement: A Secondary Analysis of a Randomized Clinical Trial. JAMA Psychiatry. 2025;82:654–62. https://doi.org/10.1001/jamapsychiatry.2025.0569. 
  - ○ Strong evidence that enhancing positive emotion regulation reduces opioid craving.
- Huang Y, Ceceli AO, Kronberg G, King S, Malaker P, Parvaz MA, et al. Association of Cortico-Striatal Engagement During Cue Reactivity, Reappraisal, and Savoring of Drug and Non-Drug Stimuli With Craving in Heroin Addiction. American Journal of Psychiatry. 2024;181:153–65.
  - ○ First identification of corticostriatal dysfunction in opioid use disorder during savoring natural rewards versus processing opioid cues.
- Lin X, Deng J, Shi L, Wang Q, Li P, Li H, et al. Neural substrates of smoking and reward cue reactivity in smokers: a meta-analysis of fMRI studies. Translational psychiatry. Nature Publishing Group UK London; 2020;10:97.
  - ○ Meta-analytic evidence of the impact of smoking on reward processing in the brain.
- LoFaro FM, Gueguen MCM, Kapoor A, Alvarez EE, Bonagura D, Konova AB. Largely Intact But Less Reliable and Distributed Neural Representations of Subjective Value in Human Opioid Addiction. J Neurosci. 2026;45.
  - ○ Evidence that opioid use disorder impacts subjective value representations.
- Meier IM, Eikemo M, Leknes S. The Role of Mu-Opioids for Reward and Threat Processing in Humans: Bridging the Gap from Preclinical to Clinical Opioid Drug Studies. Curr Addict Rep. 2021;8:306–18.
  - ○ Extensive review of the impact of opioids on reward processing.
- Mian MN, Earleywine M. Savoring as an intervention for cannabis use: acceptability, feasibility, and preliminary results. Addiction Research & Theory. Taylor & Francis; 2023;31:296–305. https://doi.org/10.1080/16066359.2022.2160871. 
  - ○ Pilot evidence that savoring can impact cannabis use.

## Data Availability

No datasets were generated or analysed during the current study.
